# Ethics Review Committee approval and informed consent: an analysis of biomedical publications originating from Sri Lanka

**DOI:** 10.1186/1472-6939-9-3

**Published:** 2008-02-11

**Authors:** Athula Sumathipala, Sisira Siribaddana, Suwin Hewege, Manura Lekamwattage, Manjula Athukorale, Chesmal Siriwardhana, Joanna Murray, Martin Prince

**Affiliations:** 1 Kings College; Institute of Psychiatry, Health Services and Population Research Department, De Crespigny Park, London SE5 8AF, UK; 2 Institutes of Research and Development, 762/4B Pannipitiya Rd, Battaramulla 10120, Sri Lanka

## Abstract

**Background:**

International guidelines on research have focused on protecting research participants. Ethical Research Committee (ERC) approval and informed consent are the cornerstones. Externally sponsored research requires approval through ethical review in both the host and the sponsoring country. This study aimed to determine to what extent ERC approval and informed consent procedures are documented in locally and internationally published human subject research carried out in Sri Lanka.

**Methods:**

We obtained ERC approval in Sri Lanka and the United Kingdom. Theses from 1985 to 2005 available at the Postgraduate Institute of Medicine (PGIM) library affiliated to the University of Colombo were scrutinised using checklists agreed in consultation with senior research collaborators. A Medline search was carried out with MeSH major and minor heading 'Sri Lanka' as the search term for international publications originating in Sri Lanka during 1999 to 2004. All research publications from CMJ during 1999 to 2005 were also scrutinized.

**Results:**

Of 291 theses, 34% documented ERC approvals and 61% documented obtaining consent. From the international journal survey, 250 publications originated from Sri Lanka of which only 79 full text original research publications could be accessed electronically. Of these 38% documented ERC approval and 39% documented obtaining consent. In the Ceylon Medical Journal 36% documented ERC approval and 37% documented obtaining consent.

**Conclusion:**

Only one third of the publications scrutinized recorded ERC approval and procurement of informed consent. However, there is a positive trend in documenting these ethical requirements in local postgraduate research and in the local medical journal.

## Background

Guidelines on ethical conduct of research have focused attention on protecting research participants [1-3]. Approval from an ethical review committee (ERC) [1,2,4] and ensuring informed consent from the participants are the cornerstones of ethical human subject research [3,5,6]. Consent is considered 'informed' when given by a person who understands the purpose and the nature of research, what is required from the participant and what may be the potential benefits and risks resulting from the study [2,7]. In order to ensure acceptable ethical standards in externally sponsored research, protocols should undergo ethical review in both the host and the sponsoring country [1,2]. However, research carried out in the developing world which does not comply with accepted ethical standards is a concern for academics, funding agencies, journal editors and governments [3].

Proof of ERC approval and consent of participants can be sought by direct and indirect methods. Inquiring from the relevant ERC or obtaining a copy of the approval is a direct method. Inquiring from the researcher or scrutinising published research for documentation is an indirect method. For informed consent, witnessing the informed consent process is the direct method whereas inquiring from the researcher, research participant, ERC or establishing evidence from documentation in published research are indirect methods.

To our knowledge there are no studies reported from South Asia examining evidence of adherence to these two requirements in published research. However, studies in South Asia have examined the issue of consent from the point of view of participants and researchers. A study carried out in Bangladesh examined participants' understanding of a community-based study of iron supplementation [8]. Although consent had been obtained after detailed explanation of the study, many participants did not understand that they were free to decline to participate, that they could choose to leave the study, and about half believed that participation was part of routine health care [8]. In another questionnaire survey among researchers in the developing world, the majority (62%) stated that they had obtained written informed consent [9]. Several studies have also looked into informed consent practices relevant to patient care and clinical ethics from the perspectives of physicians and patients [10-12].

Although Sri Lanka is a developing country with a population of 19 million, it has strengths in health and education [13] and the tradition of biomedicine, both western and eastern, is firmly established. There is archeological evidence of hospitals dating back to the 9^th ^century AD in the ancient kingdom of Anuradhapura [14]. The civil medical department was established by the British in 1858 and the Colombo Medical School, which opened in 1870, is the second oldest in south Asia [14]. The *Ceylon Medical Journal *(CMJ) is the oldest surviving English language medical journal in Australasia, first published as the *Journal of the Ceylon Branch of the British Medical Journal *in 1887 [14].

Sri Lanka has a long history of scientific research. Aldo Castellani, under British rule, established the Medical Research Institute (MRI) in 1901 [14]. Influential international research collaborations have been reported [15-21] and leading funding agencies, including the Wellcome Trust (UK), National Institute of Health (USA) and the World Bank have funded research in Sri Lanka. A considerable amount of anthropological and sociological research has also been conducted [22].

In spite of the volume of research, bioethics is at an early stage of development in Sri Lanka [23]. There are no formal training courses for clinical researchers except for some limited teaching in the six medical faculties from which the majority of research emanates [24]. Institute for Research and Development has conducted a basic course in 2003 and a basic and advanced course in 2007.

In this study we draw attention to the issue of informed consent and ERC approval for human subject research in contemporary Sri Lanka. The International Committee of Medical Journal Editors (ICMJE) guidelines of 1981 required researchers to indicate that the research had IRB review [25] and, in 1991, the ICMJE added that when "informed consent has been obtained by authors, this should be clearly stated in the article"[26]. This study aimed to determine the extent to which ERC approval and informed consent procedures were documented in three independent sources of publications arising from human subject research carried out in Sri Lanka.

## Methods

The protocol was revised in accordance with the recommendations of reviewers of the funding body. Consensus generation meetings to fine tune the protocol were conducted with the participation of the authors and those who were trained during the ethics course and qualitative workshops conducted by the Institute for Research and Development [27].

### Data collection

1. All published MD and MSc theses from 1985 to 2005 available at the Postgraduate Institute of Medicine (PGIM) library affiliated to the University of Colombo were scrutinized. PGIM is the only institute in Sri Lanka that awards post graduate medical degrees. Details of ERC approval, information leaflets and consent forms used in the research conducted to obtain the postgraduate degrees were recorded. The theses available at the PGIM were initially scrutinized by two authors (ML and MA) to ensure a comprehensive coverage. Later another two authors (SH and CS) checked the list against the bibliography of theses published by the PGIM. Then theses were perused by the two authors (ML and MA) based on pre-agreed checklists to identify and record

• any information relating to ethical approval in the methodology or elsewhere in the published work.

• If yes,

(a) Whether the ERC which granted ethical approval was named and

(b) Whether any evidence was provided; e.g.: copy of the ethical approval letter

• Information on the type of consent obtained (written/oral) and methods used in obtaining consent

• Whether a copy of the information leaflet given to the participant was attached

• Whether a copy of the consent form was attached to the thesis.

2. A Medline search was carried out with MeSH major and minor heading using 'Sri Lanka' as the search term to find all published research originating from Sri Lanka, between 1^st ^January 1999 and 1^st ^September 2004. We downloaded full text papers published in open access journals and those available in Athens, an access management system of academic articles. If the full text papers were not available we downloaded the abstracts. We could not obtain the full text paper copies of those abstracts as the printed journals were not available in Sri Lanka. These publications were initially scrutinized by two authors (ML and MA). Later AS, SS, CS re-scrutinized the papers to select and agree on human subject research publications. Then AS and SS independently scrutinised the papers to identify

• any information related to ERC approval in the published work

• documentation on the type of consent obtained (written/oral)

3. For CMJ we analyzed the full text papers available as hard copies for the period 1999–2005. This was due to two reasons; it is the only Sri Lankan journal available in the PubMed as abstracts and we had access to hard copies. CMJ publications were initially scrutinized by two authors (ML and MA). Later CS and SH re-scrutinized the papers to select human subject research publications and to identify whether ERC approval and consent was documented.

## Results

### Survey of research theses

A total of 305 MD and MSc theses were available at the PGIM library from 1985 to 2005. Of these 291 (95.4%) concerned human subject research. Eighty seven (29.5%) were submitted by candidates for the three research based postgraduate qualifications at the PGIM, namely MD in Community Medicine, Community Dentistry or Family Medicine. Overall 99 (34%) theses had documented ERC approval. However, only 20(6.9%) provided evidence. One hundred and seventy eight (61.2%) theses had documented obtaining consent, and 12 stated that informed consent was not relevant. The majority of the above these (67; 68%), had obtained approval from the University of Colombo-Medical Faculty ERC, while 8 (8%) had obtained approval from Medical Research Institute ERC, 7 (7%) from Kelaniya University ERC, 6 (6%) from Peradeniya University ERC, 6 (6%) from Sri Jayawardenepura and 4 (4%) from Ruhuna Medical Faculty ERC. See table 1.

**Table 1:** Documentation of ERC approval and consent in different fields of study

	**Documentation of ERC approval (%)**	**Documentation of obtaining consent (%)**	**Total**
MD Community Dentistry	4 (100)	4 (100.0)	4
MD Community Medicine	35 (48.6)	32 (44.4)	72
MD Microbiology	14 (48.3)	21 (72.4)	29
MD Family Medicine	5 (45.5)	7 (63.6)	11
MD Gyn & Obs	7 (36.8)	16 (84.2)	19
MSc Community Medicine	31 (23.8)	81 (62.3)	130
MSc Community Dentistry	3 (13.6)	14 (63.6)	22
Medical Administration	0	3 (75.0)	4

Total	99(34.0)	178 (61.2)	291

Most theses (130; 44.7%) were submitted by candidates applying for MSc in community medicine. However, the candidates for MD community medicine had more frequently documented ERC approval (23.8% of MSc candidates compared with 48.6% of MD candidates). The same trend can also be seen in community dentistry albeit in smaller numbers (13.6% of MSc candidates compared with 100% of MD candidates). See table 2.

**Table 2:** Documentation of ERC approval and consent in theses; five yearly trends

**Theses by 5 year period**		**Documentation of ERC Approval**		**Documentation of obtaining consent**
**Year of submission**	**Total**	**Documented (%)**	**Approval letter attached (%)**	**Documented or not relevant (%)**

1980 – 1985	09	0 (0)	0 (0)	3 (33.3)
1986 – 1990	33	4 (12.14)	0 (0)	10 (30.0)
1991 – 1995	91	15 (15.4)	1 (1.1)	52 (57.1)
1996 – 2000	69	36 (52.1)	5 (7.2)	50 (72.5)
2001 – 2005	89	44 (49.4)	14 (15)	71 (79.8)

Total	291	99 (34.0)	20 (6.9)	186 (63.9)*

* 8 thesis have mentioned that consent was not relevant

Documentation of ERC approval first appeared in a thesis submitted in 1989 and evidence of approval was attached for the first time in 1995. Table 3 (columns 3 & 4) shows an increasing trend of candidates documenting ERC approval and providing evidence by annexing a copy of the approval letter. Also during the last 10 years, 71% of researchers have documented obtaining consent from the participants for their research (column 5). More candidates documented obtaining consent from participants than ERC approval (63% compared to 34%).

**Table 3:** The manner how consent is described when it was documented; 5 yearly trends

	**Consent**			**Informed consent**		
	
	**Not specified**	**oral**	**written**	**Not specified**	**oral**	**written**
1980 – 1985	2	0	0	0	0	0
1986 – 1990	4	2	0	2	0	1
1991 – 1995	17	5	3	14	2	8
1996 – 2000	11	3	8	16	1	9
2001 – 2005	8	5	4	20	14	19

Total	42	15	15	52	17	37

However, only three (1%) information leaflets and 23 (7.5%) consent forms were available from 291 theses.

## Survey of published research in peer reviewed journals

### Medline search results

The Medline search carried out for the study identified 367 publications (excluding CMJ publications), originating from Sri Lanka during the period from 1st January 1999 to 1^st ^September 2004. Of these 250 concerned human subject research. We were able to download 111 full text papers and the remaining 139 were available only as abstracts. Of the available 111 publications on human subject research, we included only original research papers and excluded 32; letters, editorials, and data arising outside Sri Lanka from Sri Lankan authors. Of remaining 79 papers only 30 (38%) full text publications had documented ERC approval and 31(39%) informed consent.

We attempted to find out how many of these papers had authors of Sri Lankan origin but it was not feasible from the information available in the papers.

### CMJ search results

CMJ is indexed in Medline but only the abstract appears. Hence they were not included in the above 79 papers. A separate analysis was undertaken of the papers published in the printed version of the CMJ during the period 1999–2005. A total of 113 papers were published but 13 were not concerned with human subjects. See table 4.

**Table 4:** Ceylon Medical Journal survey; ERC approval and consent

**Year of submission**	**ERC approval documented (%)**	**Documentation of Obtaining consent (%)**	**Not Relevant (%)**	**Total**
1999	2 (15.4)	5 (38.5)	0 (0)	13
2000	3 (23.1)	2 (15.4)	2 (15.4)	13
2001	5 (31.3)	3 (18.8)	0 (0)	16
2002	5 (41.7)	5 (41.7)	0 (0)	12
2003	6 (37.5)	3 (18.8)	4 (25)	16
2004	6 (46.2)	7 (53.8)	1 (7.7)	13
2005	9 (52.9)	12 (70.6)	1 (5.9)	17

**Total**	36 (36)	37 (37)	8 (8)	100

Two (15.4%) of the 13 papers published in CMJ in 1999, had documented obtaining ERC approval. By 2005 this had increased to 9 (52.9%). In 1999, five (38.5%) of the original articles published in CMJ had documented obtaining consent from the participants. In 2005, this had risen to 12 (70.6%) with eight (44%) specifying 'informed consent'.

## Discussion

The survey results of three sources of research publications in Sri Lanka, theses, international journals and CMJ, all show an increasing trend in documenting ERC approval and participant consent.

Although from 1986 to 1990 only 12.4% had obtained ERC approval, this increased to 49.4%, during 2001 to 2005. More specifically, reporting about ERC approval increased from 12.5% in 1989, to 75% in 2005. Documentation of consent increased from 30% to 79.8% during the same period. It also emerged that candidates for higher qualifications (MD as opposed to MSc) documented ERC approval at a higher rate in their research projects and publications.

There is no reference to ERC approval or consent in PGIM examination rules and regulations. However each board of study is responsible for ensuring these standards. Board of study in community medicine has made it a requirement that candidates obtain ERC approval (personal communication from the chairman) since 1991. Regulations and guidelines of the board of study in family medicine state that research protocols should include details of approval of the project by a relevant ERC. We were unable to find information about requirements for ERC approval in other disciplines. Instructions to authors in CMJ [28] state that ethical committee approval should be mentioned in the text in 'intervention studies' with a photocopy of the approval letter attached to all submissions.

Only 38% of full text papers from 1999 to 2004 in international journals had documented ERC approval. This is a cause for concern. In CMJ, ERC approval for the same period was 32.5%. During the same period 39% of papers in international journals and 30.1% in CMJ had documented informed consent.

However, absence of documentation of ERC approval cannot be taken as absence of obtaining approval, as some may have not reported it. Further research should include direct inquiries to authors and journal editors on ERC approval for published research.

Even though the International Committee of Medical Journal Editors requires ERC approval and informed consent to be documented in the manuscripts [25,26], our results shows that this requirement has not been strictly adhered to. Previous studies that had examined research carried out in the developed world have also shown that documentation of IRB review and IC is not consistent, even in journals that state it as a requirement [29-34]

### Limitations

This study relies on self reported data. Therefore, the content of the ethical review, the process of informed consent, and the level of understanding of research subjects, both of the research, the consent process, and the meaning of informed consent are not explicitly stated. This affects all three surveys. In addition the main limitation in the international journal survey is the inability to review full publications on human subject research as only the abstracts were avaialble. Given space limitations, it is reasonable to anticipate that ethical review would not always be stated in the abstract.

In all three surveys, it is possible that more researchers may have sought ERC approval than reported. Evaluation based on documentation by the researchers as opposed to actual ERC approval and consent is an inherent weakness of the study methodology.

## Recommendations

Researchers should be requested to attach the information leaflet and consent forms to the final version of their theses, in the same way they are required to append research instruments. This will enable the examiners to assess adherence to good ethical practices and encourage new researchers to regard ethical standards as an integral part of high quality research. Similarly journal editors should implement ICMJ recommendation more robustly.

## Conclusion

Documentation of ERC approval and consent occurs in around 35% of research publications in international journals and CMJ. Even though there is a positive trend towards documentation of ERC approval and informed consent in human subject research in Sri Lanka, the prevailing situation is not satisfactory. Increasing awareness on these two crucial safeguards; ERC approval and informed consent, will be helpful locally to promote basic ethical standards as essential components of scientific research. This may also help adherence to higher ethical standards in international collaborations.

## List of abbreviations

PGIM- Postgraduate Institute of Medicine, ECR- ethics review committee, CMJ- The Ceylon Medical Journal, MRI- Medical Research Institute, MeSH-Medical subjects headings

## Competing interests

AS is a Sri Lankan academic based at IoP – UK, and also at IRD – SL, both honorary positions at the time of conducting this research as well as at present. None declared by others.

## Authors' contributions

AS proposed the initial conceptual framework for the research and was responsible for overall conduct of the project. AS, SS, SH, MP and JM were involved in the protocol design, analysis and or interpretation of data, writing and editing the manuscript. SS. SH, ML, MA, CS contributed to the collection and analysis of data including the statistical analysis.

AS prepared the first draft. SH, CS and SS wrote the second draft. All authors were involved in subsequent editing and agreed on the final version.

## Pre-publication history

The pre-publication history for this paper can be accessed here:


